# Emphasizing the understanding of social norms for cross-cultural public health behavioral interventions

**DOI:** 10.3389/fpubh.2026.1714896

**Published:** 2026-04-30

**Authors:** Hatem H. Alsaqqa, Abdallah Alwawi

**Affiliations:** 1 Public Health Department and Deanship of Scientific Research, Al-Quds University, Jerusalem, Palestine; 2 Palestinian Ministry of Health, Gaza, Palestine; 3 Nursing Department, Faculty of Health Professions, Al-Quds University, Jerusalem, Palestine; 4 INTI International University, Nilai, Negeri Sembilan, Malaysia

**Keywords:** culture, health promotion, intervention, public health, social norms

## Abstract

The concept of social norms has been extensively employed in behavior-change-oriented public health interventions. By addressing these problems, health programs that use social norms may become more rooted in the complex social realities that influence behavior. Nonetheless, researchers of global health have shown a greater interest in altering social norms as a means of advancing health and wellbeing in nations. Despite this growing interest, the ability to apply social norm theory to guide health interventions varies greatly. Understanding the function of social norms in public health interventions across contexts is the main goal of this point of view essay. Understanding how social norms change over time can help us better understand what works to support people in leading change in public health interventions, as social norms have been shown to be an effective way to modify population behavior and improve health outcomes. Several interventions can improve the efficacy of their treatments by being aware of these issues. However, the evaluation and development of the Social Norms Approach will be aided by a more thorough assessment of social norms-based interventions and increased methodological rigor.

## Highlights

- Understanding the function of social norms in public health interventions across contexts.
- Understanding how social norms change over time and what works to support people in leading change in public health interventions.
- Evaluation and development of the Social Norms Approach aided by assessment of social norms-based interventions in public health.
- Social norms interventions have a substantial impact on clinical behavior and the ensuing health outcomes of patients.
- Program planners must comprehend the social norms surrounding the behavior of interest, and the facilitators to effectively implement them.

## Introduction

Theoretically, social norms have an impact on behaviors connected to health, like eating habits and physical activity. Many theories of health behavior, such as the Social Cognitive Theory (1) and the Theory of Planned Behavior (2), have included the widely held belief that social norms play a significant role in determining healthy behaviors. The literature demonstrates a variety of methods for examining cultural dynamics in social normative impact. Comparative study designs, in which data from a U. S. sample are compared to a sample or samples of people from another country, have been used in many of these studies (3). Research employing these ideas occasionally provide proof of the validity and measurement reliability of the study measures using sample data [e.g., (4)].

However, social norms are the unwritten, informal guidelines that specify what is proper, acceptable, and required behavior in a certain group or community. Cialdini and colleagues’ work, which defined social norms as one’s ideas about (1) what others in one’s group do (descriptive norms) and (2) what they accept and disapprove of (injunctive norms), is largely responsible for interest in social norms theory among contemporary practitioners (5, 6). Empirical evidence has shown the influence of norms. Cialdini’s concepts have been used by researchers to show how social norms affect a number of health-related behaviors, such as drinking alcohol (7, 8), eating food (9), using drugs recreationally (10), smoking (11), purifying water (12).

An overall understanding that no paradigm of social conduct can be regarded as a phenomenon-in-itself leads to the treatment of social norms as contextualized and culturally embedded. Therefore, research on civility has focused on Western countries, whereas collectivism and authority are more important in Eastern cultures. Everything is ingrained, albeit to varying degrees, because ideas of culture and its ideational and practical appearances are inherently holistic and integrated.

Yet in their later research, Rimal and Lapinski (13) place norms in a variety of behavioral rules that encompasses both what are more popularly referred to as norms and codified laws and traditions. According to Rimal and Lapinski (13), the distinguishing characteristic is that norms are “socially negotiated and contextually dependent. However, the division of social norms into descriptive and injunctive norms was also proposed by Cialdini and colleagues (5). The former raises behaviors that are frequently performed (the “is”), while the latter raises actions that are thought to be accepted or disapproved of (the “ought”) by members of a reference group.

Group identification plays a crucial role in explaining how normative misperceptions impact behavior, particularly when examining which normative messages are most pertinent and effective in persuading the target audience. Theoretically, social identity methods emphasize how self-categorization contributes to conformity to group norms (14). In general, people who identify firmly with a particular social group are more inclined to follow its standards, both as a guide for their own behavior and in part as a means of fitting.

Moreover, the relationship between norms and values is not linear or static. Norms may endure when values shift because they are a representation of social behavior. According to Edberg’s description of narcotraffickers, norms as fluent symbols can also manifest as the performance of particular social roles, such as the regularized behavior patterns linked to gender roles or the enactment of a cultural persona (15). Norms can enact and uphold power structures, and they can be connected to power outlines as codes or behavioral standards. For instance, bowing or prostrating oneself in front of a king or emperor used to be customary. This standard is obviously a manifestation of a power imbalance (16).

Norms are dynamic and usually evolve over time for a variety of reasons, independent of any social or behavior change interventions. Our comprehension of how communities uphold and transmit dominant social practices depends heavily on the function of social norms (17). In this way, norms “solve” an issue with group coordination. Furthermore, according to Boyd and Richerson (18), norms change when “societies shift from one coordination equilibrium to another.” Similarly, norms have been defined as behavioral decisions that optimize the effective achievement of societal objectives (19).

Nonetheless, social network theorists have also conceived social norms as relating to networks and reference groups, shifting “outward” from an emphasis on the rational individual. As a particular kind of innovation diffusion, normative change is explained by network dynamics, which include influencers’ activities and communication through networks of persons, where the innovation is a pattern of conduct (20).

In essence, norms are a collection of behavioral options that people as socialized actors assimilate and find desirable, according to Parsons’ formulation (21). Other members of a society share the specifics of this behavioral set, which forms its value system and is a prerequisite for social unity. According to Tajfel and Turner (22), social norms are also considered indicators of group identification and affiliation, with members of a group frequently embracing them as part of their self-categorization.

As a result, they offer instructions for interpretation that are usually linked to behavioral scripts.

Cultural paradigm (consensus), the extent to which members of the group possess in-depth knowledge of the model (competence), and the extent to which individual actions or behaviors, even when shared, truly follow the model (consonance). Research by Dressler and colleagues (23) has shown the health ramifications of cultural models and the variance that goes along with them. For instance, there is a correlation between high blood pressure and low cultural consonance, which is the difference between a person’s actual lifestyle and culturally shared models of an appealing one (16).

## The relation between behavior and social norms

According to several well-known psychological theoretical models of health behavior, social norms are significant drivers of health-related behaviors (2, 24). According to ideas in social and health psychology, there are several ways to explain how social norms influence behavior. According to Cialdini and Trost (25), social norms are typically defined as guidelines and standards that members of a society understand, without the force of legislation, direct or restrict social conduct. These members regularly have to work with the supposed social pressure to engross in or refrain from engaging in particular behaviors (2). Social norms usually function implicitly, use people’s perceptions of acceptable behavior to influence behavioral patterns and intents, even though they can be based on direct and public communication between group members (14).

Despite the fact that there are various methods to construct social norms (26, 27), perceived normative peer behaviors and attitudes have become important indicators of health-related behaviors. Perkins and Berkowitz (28) and McAlaney et al. (29) have provided evidence that people may have trouble estimating the true behavioral and attitudinal norms of their peers and associated social groupings. One significant effect of these social norm misunderstandings, also known as “normative misperceptions,” is the possibility of engaging in hazardous behaviors because one mistakenly believes that these behaviors are typical of one’s peer group (29).

The emphasis on modifiers between behavior and norms, describing their routes and the circumstances in which they function, is significant in this work (27). Salience, or the degree to which a certain norm is emphasized or activated in a given social context, the availability of particular attitudes about a norm in a given social context, and social modeling of behavior—the process through which a person exhibiting a behavior is perceived and treated favorably, as in Bandura’s construct of a social model that is the foundation of his Social Learning Theory (SLT), which holds that people pick up new attitudes, behaviors, and emotional responses by watching, copying, and modeling others (which are a few examples of these moderators) (13, 27).

However, the “Social Norms Approach” (SNA) is a behavior modification technique that was developed in response to evidence that people frequently misinterpret their peers’ participation in a variety of health-related behaviors, both positive and negative (30). The SNA has been a commonly used behavior modification method in addition to research on the impact of social norm misperceptions on behavior (29). By addressing these misunderstandings of social norms, SNA-based interventions seek to decrease harmful health behaviors and encourage beneficial ones (29). According to Perkins H. W. et al. (31), the SNA makes a number of assumptions regarding the impact of these social normative perceptions on behavior. These include the following: (i) that perceived norms are constantly linked to behaviors; (ii) that people frequently misperceive or overestimate the behaviors and attitudes of their peers; (iii) that these misperceptions are linked to amplified or diminished appointment in those behaviors; and (iv) that interventions that address these misunderstandings should encourage more positive behaviors.

Low views of health hazards are rendered meaningless by high perceptions of social norms, suggesting that social norms may serve as a significant lever for encouraging preventative behavior. Institutions in charge of handling the problem must therefore continue to garner and strengthen support at the social level. Information campaigns, model behavior by officials (32), and open communication about expected behavior (33) can encourage citizens to adopt the desired behavior.

Moreover, there are numerous hypotheses explaining social norms and how they affect behavior (34). Cialdini’s theory, which distinguishes two types of normative beliefs—descriptive norms, which are beliefs about what other members of one’s group do, and injunctive norms, which are beliefs about what members of one’s group approve or criticize of—is cited in the majority of the literature (27, 35, 36). Theorists contend that in order to alter a social norm, it is essential to include the complete network of people who share that norm, or to influence people’s reference group (35, 36).

Attention should be paid to how social norms are frequently decontextualized in applications of behavior and social change in relation to the social and cultural contexts in which they are ingrained and influenced. By using social norms three objectives are to: (1) provide a general overview of the various ways that the social norms construct are characterized and utilized across disciplines as pertinent to and applied in health behavior change applications; (2) suggest a set of theoretical and practical considerations regarding social norms that could be incorporated into the design and implementation of interventions related to social norms to improve the validity of social norms as a change construct, reduce the likelihood of unintended consequences, and boost the sustainability of interventions; and (3) provide a working definition that better reflects the variety of ways that norms can impact behavior (16).

Lastly, given that norms can act as symbols for underlying values or power structures, programs aimed at altering them may fall completely short if the norms have changed and are no longer useful for instantiating a specific set of values or a structural node. Or, in periods of rapid societal change, particularly when fueled by technology, when behavior has changed but norms and values have not yet followed up (cultural lag) (16).

## Dynamics and measurements of social norms

Applying theories and methods in unfamiliar cultural contexts could pose problems. Scholars of culture have proposed both qualitative and quantitative methods for studying the static features of culture because the dialectical character of culture might be difficult for researchers’ approaches to investigate the dynamic ones (37). For instance, demographic traits like religion and ethnicity can be measured as predictive variables when researching dietary practices among cultural groups.

However, essential concept in health promotion research is cultural sensitivity. It is attained by heightened awareness of cultural variations in respect to norms, beliefs, and values (38). It is characterized as “sensitivity to the characteristics of a culture or to the dynamics of a social group” (39). Any specific norm’s relationship to the underlying structure of power, implication, or social organization it symbolizes is dynamic as part of that process.

However, a review of the current research literature on the subject reveals significant variation in the conceptualization and measurement techniques of social norms (40, 41). Since the conceptualizations combine aspects of belief and processes, they have a lot in common with other notions that are more focused on processes, such as result expectations, moral standards, and social support. There is also significant variation in the operationalization of the concept of a norm within each of these normative realms.

Descriptive norms, for instance, have been evaluated in a variety of ways as the proportion of individuals thought to participate in a specific behavior. Another way to quantify injunctive norms is to rate how much people support and promote good behavior or how much they disapprove of unacceptable behavior. The specificity and complexity of behavioral definitions vary in both domains, ranging from the very general (e.g., eating a “healthy” diet) to the very specific (e.g., exercising at a criterion level of intensity for at least 30 min at a time at least 5 days a week for the last 6 months). Furthermore, the reference group used to define norms varies depending on the study; it can be individuals in general, people the respondent knows, or particular peer groups that the respondent strongly identifies with Gavin et al. (42).

Nonetheless, there are several sources of qualitative methods and approaches for diagnosing social norms (43). Factors that may either increase or decrease the impact of social norms messaging on iron folic acid use include perceptions of being at risk for developing anemia, expectations that taking iron folic acid will lead to health benefits such as preventing anemia or reducing fatigue, and the access to and availability of iron folic acid. Interventions must therefore focus on improving the social norms around a behavior as well as any potential moderators that might reinforce the link between social norms and behavior. The most successful social norms manipulations occur in one’s own context (such as a field trial), target collectivist groups, and provide messages in many formats (30).

## Change in public health interventions and social norms

It is evident that the norms construct has a more nuanced meaning and application than its usual application to social and behavioral transformation would imply. The anthropological viewpoints discussed above provide more insights on norms and how they relate to behavior, society, and culture. In fact, they summarize how norms are discussed in anthropological literature. As a result, “changing norms” as the preferred social/behavioral change tactic to enhance health interventions impact is far more challenging than it seems.

Researchers suggest that interventions that aim to connect norm changes to behavior changes and better health outcomes should use basic or formative research to determine how embedded the social norms in question are and how much the health intervention is a direct result of just one or a few chosen norms (16). This entails gauging the degree to which social norms, penalties, and collective, frequently subconscious beliefs uphold the unwritten laws that govern behavior (44).

When social judgments are important and the best course of action is unclear, norms are believed to be more prominent for conduct. In stressful situations, social evaluation demands are higher. Smith (45) conducted a recent country-level analysis that provided some support for this line of thinking. Smith discovered that in situations that Gelfand et al. (46) had categorized as more strictly structured, helpful behavior was more closely linked to descriptive norms. According to this logic, in tighter cultures as opposed to loose cultures, norms ought to be a better indicator of behavioral intentions and definite behavior.

On the other hand, cultural awareness denotes to the healthcare provider’s knowledge of the dissimilarities between themselves and others from different backgrounds, particularly with regard to variances in attitudes and values (47). It is essential to understand that ignorance can lead to bad choices and detrimental outcomes for the people it is meant to serve. Cultural sensitivity reduces the risk of making bad decisions while increasing the likelihood of making correct and suitable ones (48).

However, there are three reasons why it is important to raise awareness of culture as a variable in public health research. First, the majority of research on health promotion is carried out in wealthy nations that are seeing an extraordinary influx of immigrants from a variety of cultural backgrounds. Second, given their widespread occurrence and significant social and economic costs, lifestyle-related illnesses are a global priority for research (49). It is impossible to discuss lifestyle choices apart from people’s cultural backgrounds. Third, research on health frequently comes from researchers in wealthy, nations whose own cultural prejudices and viewpoints shape their scientific lenses.

In addition to physical capabilities, health becomes a positive concept that emphasizes social and personal properties. Three fundamental prerequisites are required for health improvements, according to the Ottawa Charter: mediation (reconciling the interests of various sectors and individuals to promote and protect health), enabling (taking action in collaboration with individuals or groups to empower them), and advocacy for health. In order to discourse the entire spectrum of potentially adjustable determinants of health, including societal status, education, employment and working circumstances, access to adequate health services, physical environments, and health behaviors and lifestyles, public health involves action and advocacy.

Moreover, individuals can use norms to further their own or their social goals and to convey messages because they can be viewed as behavioral codes, rules, common patterns, ways of legitimizing behavior, or valued patterns that are somewhat familiar to members of a cultural group. This is made feasible by the widespread knowledge of norms and their cultural significance; as such, deliberate handling or breaking of a norm serves as a social and communication tool, much like normative conformity.

This is also made possible by the fact that a variety of norms, some of which are likely to be in conflict with one another, might be applied to a particular social circumstance. Those in positions of power (such as politicians or patriarchs) or those without power but vying for it may purposefully alter norms. Normative conflict can be strategically employed to expand the social expectations for a particular social circumstance that are generated by social norms, as well as the spectrum of behavior that may be legitimated and hence suitable.

Nonetheless, social norms marketing strategies seek to rectify people’s erroneous impressions of what members of their group do and find acceptable in order to alter group behavior. Personalized normative feedback is the second tactic, in which individuals are informed about their performance in comparison to those around them.

Three processes for changing social norms were identified by Cislaghi (50, 51) after researching successful facilitator-led programs: (1) action, where participants widely implement their policies and reassure others to join the assembly, ultimately achieving the critical figure required for normative change; (2) deliberation, where contributors establish a new positive norm within their reference group and choose approaches to stimulate others in their settings; and (3) motivation, where individuals understand about the adverse impacts for themselves and others of complying with the destructive norm (52). Finally, health can be evaluated as a comprehensive notion from the standpoint of public health. According to this viewpoint, health is a complicated phenomenon, and as such, integrating various disciplines might be crucial to improving people’s health.

## Cross-cultural communication

There is variation in the values that people inside nations support, in addition to variations at the national level. Therefore, it is crucial to look at individual support for cultural values as well as contextual differences. The country level of analysis is denoted by “cultural values” or “themes,” whereas the individual level of analysis is denoted by “cultural orientations” (53).

However, social norms influence our conduct; researchers are greatly influenced by what others believe people do and find acceptable (54). This realization is not new, as scholars have been examining norms both overtly and implicitly since the late 19th century, when modern psychology, sociology, and anthropology first emerged. However, the question of whether standards work the same way in different cultures has received little attention up to this point. This raises the intriguing possibility that norms may also function inversely across cultural groups, which is slightly startling given that norms are by definition shared among group members and vary across groups [e.g., (55)]. Triandis (56) in his now-classic characterization of individualism–collectivism suggested that norms are more significant for individuals in collectivistic environments. Over the past 20 years, norms research has seen a resurgence in the field of cultural psychology. For instance, the idea of intersubjective descriptive norms [e.g., (57)] has become increasingly popular as a means of elucidating cultural differences.

Moreover, comparing occurrences from different cultures is known as cross-cultural (58). Recognizing how interactions with people from different cultures may differ from those that are typical of one’s own culture is a crucial component of a cross-cultural relationship (59). Cultural differences are crucial, and collaboration and an understanding of them are necessary for collaborative and synergistic interactions (59).

In order to accomplish common objectives and progress miscellaneous and multifaceted resolutions to health issues, cross-cultural cooperation between individuals and communities is indispensable. Young (60) distinguished four primary methods for compliance: (1) Coordination: persons track what they consider to be mutual rules for that action because they desire to accomplish a goal that calls for harmonized effort among group members; (2) Social pressure: people follow social morals even when they may not need to, because they suppose social benefits or punishment for ensuing or breaking them; they want to get the former and avoid the latter; (3) Signaling and Symbolism: they obey what they believe to be the group’s rules in order to indicate to themselves and/or others that they belong to that group; and (4) Reference and benchmark points: people internalize norms about what behavior is appropriate in a particular circumstance to the extent that they naturally adhere to them (61).

Nonetheless, one important element influencing how a person’s health and health-related behaviors evolve is their local community (62). Increasing people’s power and control over the circumstances that impact their health can be a strategic goal of public health in the local community. The local populace can be regarded as an active collaborator in local health efforts (63) since health can be impacted by the interaction of social networks, life events, and environmental factors (64). Facilitating cooperative decision-making to address shared issues and motivating the local community to take action are the primary objectives of public health (65).

## Benefits of social norms based-interventions and ethical cautions

A person or group that exposes the target to their ideals, beliefs, or actions is referred to as the reference person or group. The average approach has the drawback of giving above-average performers feedback that suggests they are already outperforming their peers, which may cause them to put in less effort (66). Social norms interventions occasionally reveal a peer benchmark, such as the top 10% of the reference group or the typical performance. Through persuasion, promoting teamwork to accomplish change, watching successful practices elsewhere, and receiving management support, behavior change treatments grounded on social norms may assist healthcare workers in overcoming obstacles to adopting suggested practices (67).

According to social comparison theory (68), people use social comparisons to assess their own skills and adopt behaviors that would align their skills with those of other members of the group. People evaluate their own group (also known as “in-group norms”) against other groups (also known as “out-group norms”), in accordance with the social identity perspective (68).

They are driven to behave similarly to the normal behavior of the group in order to maintain their social identity (as members of their “in group”). A concept included in the Theory of Planned Behavior (24) is the “subjective norm,” which denotes to a person’s incentive to conform to the ideas of others as well as their assessment of whether or not important others believe they should engage in a particular behavior.

Through a social norms intervention using descriptive norms, the target learns about the actions of other members of the reference group (54) as message for example (a nurse receiving information about nurses’ wound dressing behaviors). By communicating social approval or disapproval of a certain behavior, an injunctive norms message informs the target worker about the values, beliefs, or attitudes of the reference group (e.g., saying that colleagues disapprove of conducting unneeded tests). Applause, gratitude, approbation, praise, and approval are examples of this Dempsey et al. (30).

However, policymakers and development professionals often have a voracious appetite for information about how to alter societal norms, but they are less willing to participate in debates about the morality of doing so and the best course of action. For ethical and practical reasons, proponents of community involvement in project design and execution have long maintained that future participants should be co-designers and co-implementers of the intervention; this strategy has also been demonstrated to produce more successful interventions (69).

The second ethical concern is who determines what constructive norms the community should embrace.

Norm Correction Strategy informs the community about itself rather than attempting to address conflicts and power dynamics within it. The negative effects of treatments that address social norms are the third and last ethical point of contention. The few remaining compliers may face exclusion even if they succeed in modifying an adverse norm, particularly if the campaign uses language that unfavorably characterizes norm supporters. If the intervention encourages an action that certain segments of the population cannot execute as a norm, ethical issues may arise even if the message is presented in an advantageous manner. Nevertheless, adopting other positive behaviors may depend on certain material or institutional resources that are not available to the most vulnerable populations, even though one person may be able to change some behaviors independently of their current material or institutional context (e.g., refraining from sexually harassing someone) (70).

To overcome some of these ethical points, understanding the social life and customs of the population being served is essential to providing treatment that is culturally acceptable. Being culturally conscious enhances the likelihood that we will make the right choices and decreases the likelihood that we will make poor ones.

Furthermore, rather than focusing only on the obvious symptoms, addressing the underlying social, economic, and cultural elements that support the continuation of detrimental social norms might assist in addressing the underlying causes. This implies that programming efforts should be clearly linked to social norm change interventions.

Nonetheless, when developing and putting into practice interventions to alter social norms, ethical and participatory program design is advised. Finding social norms and reference groups to guide the intervention’s design can be facilitated via formative research.

## Conclusion

Social norms interventions have the ability to be implemented widely and have a substantial impact on clinical behavior and the ensuing health outcomes of patients, despite the comprehensive outcome being modest and very variable. Social assessment worked especially well when paired with cues and prompts. It was discovered that these interventions worked well in a number of settings and delivery methods. Program planners must comprehend the social environment, the social norms surrounding the behavior of interest, and the facilitators and barriers at various levels of the socio-ecological model in order to effectively implement them. Therefore, in their efforts to control public behaviors, governments and decision-makers may achieve better outcomes by emphasizing the significance of social cohesion and integrity.

Nonetheless, cultural influences must be considered when creating public health programs since they have a noteworthy impact on lifestyle choices, health perception, and health-seeking behavior research. Public health policymakers would combine reflections on efficacy with deliberate ethical action, even though creating successful interventions is crucial. Lastly, the interface between health care and cultural aspects should help future research. This indicates a rich and fruitful collaboration between academics, anthropologists, sociologists, and others in cross-cultural studies in the search for best practices.

## Data Availability

The original contributions presented in the study are included in the article/supplementary material, further inquiries can be directed to the corresponding author.
